# EGFRvIII Mediates Hepatocellular Carcinoma Cell Invasion by Promoting S100 Calcium Binding Protein A11 Expression

**DOI:** 10.1371/journal.pone.0083332

**Published:** 2013-12-20

**Authors:** Xiaoying Luo, Hailong Xie, Xiaolan Long, Min Zhou, Zhibin Xu, Bizhi Shi, Hua Jiang, Zonghai Li

**Affiliations:** 1 State Key Laboratory of Oncogenes & Related Genes, Shanghai Cancer Institute, Renji Hospital, Shanghai Jiaotong University School of Medicine, Shanghai, China; 2 Cancer Research Institute, University of South China; Hengyang, Hunan, China; University of Hong Kong, Hong Kong

## Abstract

Epidermal growth factor receptor (EGFR) is frequently aberrantly expressed in cancer, and abnormal signalling downstream of this receptor contributes to tumour growth. EGFR variant III (EGFRvIII) is the most commonly altered form of EGFR and contains a truncated ligand-binding domain. Aberrant signalling downstream of this receptor contributes to tumour invasion. We previously reported that EGFRvIII can promote hepatocellular carcinoma (HCC) invasion. However, little is known concerning the mechanisms underlying EGFRvIII-mediated increases in cell motility and invasion in HCC. In this study, we observed that S100A11 was significantly upregulated in Huh-7 cells that overexpressed EGFRvIII. Moreover, S100A11 expression was elevated in HCC tissue samples (68.6%; 35/51), and this elevation was correlated with EGFRvIII expression (*p* = 0.0020; n = 20). Furthermore, the overexpression of S100A11 can promote HCC cell invasiveness, whereas siRNA against S100A11 can suppress the invasiveness of HCC cells stably transfected with EGFRvIII. Additionally, STAT3 inhibitors can block S100A11 expression and S100A11 promoter activity in HCC cells with stable overexpression of EGFRvIII. Furthermore, mutation in STATx binding sites could abolish the S1000A11 promoter activity stimulation by EGFRvIII. Taken together, the results demonstrate that the EGFRvIII-STAT3 pathway promotes cell migration and invasion by upregulating S100A11.

## Introduction

HCC is the sixth most common cancer and the third most common cause of cancer mortality worldwide. Hepatocellular carcinoma (HCC) is a highly aggressive tumour that is rapidly fatal [1]. This type of cancer is usually diagnosed at a stage when the disease is already advanced and incurable. Tumour recurrence after a curative liver resection is high, although surgery is the most effective treatment for HCC [2], [3].

Epidermal growth factor receptor (EGFR) is a 170-kDa transmembrane glycoprotein that belongs to the receptor tyrosine kinase family of growth factor receptors. Due to its important contributions to tumour cell survival, proliferation, and motility, EGFR has been associated with many human malignancies, such as breast cancer, lung cancer, brain cancer, prostate cancer, and liver cancer[4]–[10]. The overexpression, deletion, and mutation of the EGFR gene are the most common mechanisms by which EGFR exerts its influence on tumourigenesis[11]–[13].

Coding sequence alterations of EGFR are frequently found in many types of human tumours[14]–[17]. In most cases, the EGFR variants are likely to be generated through genomic deletion. Conversely, in some instances involving the deletion or rearrangement of the intact exon(s), variants may occur as a consequence of alternative splicing[18]. The most common EGFR variant is the type III EGFR deletion mutant (EGFRvIII), which has an in-frame deletion of exons 2 to 7. EGFRvIII has been detected in 16% of non-small cell lung carcinoma cells, 57% of high-grade gliomas, 24% to 67% of glioblastomas, and 42% of head and neck squamous cell carcinomas[19]–[21].

EGFRvIII expression has also been detected in HCC tissues[22], cell lines [10], and the serum of HCC patients[23]. EGFRvIII expression can promote tumour cell migration and invasion[24]–[26]. To explore the molecular mechanisms by which EGFRvIII promotes cell migration and invasion, we report here that S100A11 is a molecular target of the EGFRvIII-STAT3 pathway in HCC.

## Materials and Methods

### Patient and tissue microarray

Participants from whom samples were obtained all provided written informed consent to participate in the study. The Ethics Committee of Shanghai Cancer Institute approved the present study as well as the consent procedure. The tissue microarray study was approved by the Ethics Committee.

Paired tumour liver tissue and adjacent non-tumour liver tissue were collected from patients who underwent curative surgery for HCC at Qidong Liver Cancer Institute, Qidong Tumor Hospital (Jiangsu, China). A diagnosis of HCC was confirmed by histological examination. The relevant clinical and pathological information was retrieved from the hospital database.

Glass slide tissue arrays of HCC were purchased from Shanghai Outdo Biotech Co. (Shanghai, China), and then, immunostaining was performed on these tissue array slides using S100A11 (ab55699; 1:50; Abcam, Cambridge, MA). Assessment of the staining was based on the percentage of positively stained cells and the staining intensity using Image-Pro Plus 6.0 software (Media Cybernetics, Inc., Bethesda, MD).

### Cell culture

The human hepatocellular carcinoma cell lines Huh-7 (ATCC), Huh7-EGFR (Huh-7 cells with exogenous EGFR overexpression) and Huh7-EGFRvIII (Huh-7 cells with exogenous stable overexpression of EGFRvIII) were constructed previously[27]. SMMC-7721 cells (with endogenous stable expression of EGFRvIII[28], Chinese Academy of Science, Shanghai, China) were maintained in DMEM supplemented with 10% foetal bovine serum in a humidified atmosphere of 95% air and 5% CO_2_ at 37°C.

### Sample preparation for two-dimensional gel electrophoresis (2-DE)

Cultured Huh-7-EGFR and Huh-7-EGFRvIII cells were harvested, and then, the cell pellets were dissolved in lysis buffer (4% CHAPS, 8 M urea, 0.5% pharmalyte, 40 mM Tris-HCl, 1% DTT, 1 mg/ml leupeptin, 5 mM PMSF and 1 mg/mL aprotinin). After 1 h of lysis on ice with gentle vortexing at 15-min intervals, the mixture was centrifuged at approximately 15000×*g* for 40 min at 4°C.

### 2-DE and image analysis

As described in the 2-DE handbook, 2-DE was performed using the PROTEAN IEF and PROTEAN II xi systems (Bio-Rad, Hercules, CA, USA). Total protein (350 µg) was solubilised in 350 µl of sample buffer (8 M urea, 2% CHAPS, 0.5% IPG, 18 mM DTT, and pH 3–10 biolytes). The IPG strips were covered with mineral oil (BioRad) and re-hydrated overnight (8000 V at 20°C). Following IEF separation, the strips were equilibrated with buffer I (6 M urea, 30% glycerol, 2% SDS, 1% DTT (pH 8.8), 50 mM Tris–HCl) and then buffer II (DTT was replaced with 2.5% IAA (pH 8.8), 50 mM Tris–HCl) for 15 min each. The equilibrated strips were individually embedded into the tops of 15% SDS-PAGE gels. SDS-PAGE was performed for 45 min at a constant power of 5 W/gel and then 10 W/gel until the bromophenol blue dye reached the bottom of the gels. The analytical gels were stained with silver nitrate, and the preparative gels were stained with Coomassie brilliant blue. The stained gels were then scanned using an Image Scanner and analysed using ImageMaster 2D Platinum 5.0 software (GE Healthcare Bio-Science, Little Chalfont, UK).

### In-gel digestion and mass spectrometry (MS)

After washing with Milli-Q water, the gels were cut with a clean scalpel to excise the relevant protein spots. The protein spots of interest were those proteins differentially expressed by more than two fold. Each slice was cut into small pieces and placed into an Eppendorf tube. The gels were de-stained twice with 50 µl of de-staining solution (25 mM NH_4_HCO_3_, 50% CAN) at 37°C for 15 min. The gels were washed twice with 50 µl of methyl cyanide and then dried at 40°C for 20 min. The gels were then pre-incubated in 18 µl of trypsin solution (12.5 ng/µl trypsin, 25 mM NH_4_HCO_3_) at 4°C for 20 min. Fifteen microlitres of 25 mM NH_4_HCO_3_ was then added to cover each gel, and the gels were incubated at 37°C for 12 h. The tryptic digests were extracted using Milli-Q water, followed by two extractions with 50% CAN/5% TFA for 1 h each. The combined extracts were dried in a vacuum concentrator at room temperature.

RP-HPLC was performed using a Surveyor LC system (Thermo Finnigan, San Jose, CA) on a C18 column 5 (0.15 mm×150 mm, BioBasic® RP-C18, 5 µm, Thermo Hypersil-Keystone). The pump flow rate was split 1∶120 to achieve a column flow rate of 1.5 µL/min. The mobile phase used was as follows: A, 0.1% formic acid in water, pH 3.0; B: 0.1% formic acid in ACN. The tryptic peptide mixtures were eluted using a gradient of 2–80% B over 60 min.

The mass spectral data were acquired on an ESI IT ion trap mass spectrometer (LCQ Deca™ XP, Thermo Finnigan, San Jose, CA) equipped with an electrospray interface operated in the positive ion mode. The temperature of the heated capillary was set at 170°C. A voltage of 3.3 kV applied to the ESI needle resulted in a distinct signal. Collision energy was automatically set by the system. After acquisition of full-scan mass spectra, three MS/MS scans were acquired for the next three most intense ions using dynamic exclusion. The acquired MS/MS spectra were automatically searched against a protein database for human proteins (SWISSPROT/TrEMBL proteome set for Homo sapiens, 12/11/2003 released) using the TurboSEQUEST program in the BioWorks™ 3.0 software suite. An accepted SEQUEST result had to have a ΔCn score of at least 0.1 (regardless of charge state). The protein identification criteria used here were based on Delta CN (≥0.1) and Xcorr (one charge≥1.8; two charges≥2.5; three charges≥3.75).

### Real-time quantitative RT-PCR for messenger RNA expression

Total RNA was extracted with TRIzol reagent (Invitrogen, Carlsbad, CA) according to the manufacturer's protocol. cDNA was reverse transcribed from 1 µg of RNA using a SYBR®Prime Script™ RT-PCR kit (Takara Biochemicals, Tokyo, Japan). The reactions were performed in an ABI PRISM®7900HT Real-Time PCR System. The thermal cycle conditions were as follows: one cycle of 95°C for 15 s, followed by 40 cycles of 95°C for 5 s and 60°C for 30 s. Each experiment was carried out in a 20-µl reaction volume containing 10 µl of SYBR® Prime Ex Taq™II (2×), 0.8 µl of forward primer and reverse primer (10 µM each), 0.4 µl of ROX Reference Dye or Dye II (50×), 2 µl of cDNA, and 6 µl of H_2_O. β-Actin was chosen as an internal control. The quantitation of mRNA was calculated by the comparative Ct (the threshold cycle) method using the following formula: Ratio = 2^−ΔΔct^ =  2^−[ΔCt(sample)−ΔCt(calibrator)]^. ^Δ^Ct is equal to the Ct of the target genes minus the Ct of the endogenous control gene (β-actin). The sequences of the primers used are shown in Table S1 in File S3.

### Western blot analysis

Immunoblotting experiments were performed according to standard procedures. The following antibodies were used: S100A11 (ab55699; 1∶1000; Abcam), STAT3 (124H6) (9139, 1∶1000, Cell signal, Boston, MA) and phospho-STAT3 (Tyr705) (D3A7:9145s; 1∶1000; Cell signal), and GAPDH antibody was used as the internal control (kc-5G4; 1∶2000; Kang-Chen Bio-tech, Shanghai, China).

### Construction of lentiviral vectors and transduction of Huh-7 cells

The complete coding sequence of the S100A11 gene was acquired by RT-PCR and then subcloned into the pWPT vector (a generous gift from Dr. T. Didier, University of Geneva, Geneva, Switzerland) to generate the lentiviral expression vector pWPT-S100A11 with *Mlu*I/*Not*I digestion. The DNA sequence was confirmed by sequencing. Recombinant lentivirus was produced in 293T cells following the cotransfection of 20 µg of pWPT-S100A11 or pWPT-GFP with the packaging plasmids (15 µg of psPAX2 and 5 µg of pMD2.G) using a calcium phosphate transfection system. The medium was changed 8 h later, and the lentivirus was harvested after 48 h. The recombinant lentiviruses encoding either S100A11 or GFP (1–3×10^6^) were used to infect Huh-7 cells (1–3×10^5^), respectively. Next, 6 µg/ml of Polybrene (Sigma, St. Louis, MO) was added to the medium.

### Silencing genes in SMMC-7721 or Huh7- EGFRvIII cells

Cells (3×10^5^) were plated on 6-cm-diameter plates and transfected 20 h later with 200 pmol of anti-S100A11, anti-STAT3, or control siRNA (Shanghai Gene Pharma Co, Ltd) using Lipofectamine 2000 according to the manufacturer's guidelines (Invitrogen). The siRNA sequences are shown in Table S2 in File S3. The effect of gene silencing by siRNA was determined by Western blotting analysis.

### Migration and invasion assays

Cell migratory and invasive abilities were assessed by the transwell migration assay and Matrigel invasion assay (BD Biosciences, Franklin Lakes, NJ), respectively. Transfected Huh-7 cells were seeded at 5×10^5^ for invasion assays and 1×10^5^ for migration assays. The invaded or migrated cells on the lower surfaces of the inserts were fixed in 4% paraformaldehyde and stained with crystal violet (Sigma) before the inserts were mounted on glass slides. More than 8 views/insert were analysed under a light microscope (200×), and the number of invaded and migrated cells was recorded. Each experiment was performed on duplicate inserts, and the mean value was expressed as a percentage from three independent experiments.

### S100A11 promoter construction and luciferase assay

The S100A11 promoter -2146/+247(+1 at ATG) was generated by PCR using genomic DNA isolated from the human HCC cell line Huh-7. The fragments −2146/+247 and −1712/+247 were inserted at the Xho I/Hind III and followed by a luciferase gene in pGL3-Basic (Promega, Madison, WI, USA). Cells were washed with culture medium containing 10% FBS and resuspended in nucleofector solution V at a concentration of 10^6^ cells/sample. The cells were transfected with 5 µg of each test construct using AMAXA nucleofector (Amaxa, Koeln, Germany) according to the manufacturer's protocol. A plasmid carrying the Renilla luciferase gene under the control of the human CMV promoter was introduced to normalise transfection and cell lysis efficiency. After transfection for 24 h, cell lysis and the determination of luciferase activity were conducted using a Dual-Luciferase Assay Kit (Promega) according to the manufacturer's instructions. Luminescence was measured using a luminometer (Berthold, Postfach, Germany).

### Statistical analysis

All experiments were repeated three times. Data are presented as the mean ± standard deviation (SD) and were analysed by Student's *t*-test. P values less than 0.05 were considered statistically significant. Statistical analyses were performed using GraphPad Prism 3.02 (GraphPad Software Inc., San Diego, CA).

## Results

### Differential expression of candidate protein between Huh7-EGFR and Huh7-EGFRvIII cells

**Figure 1:**
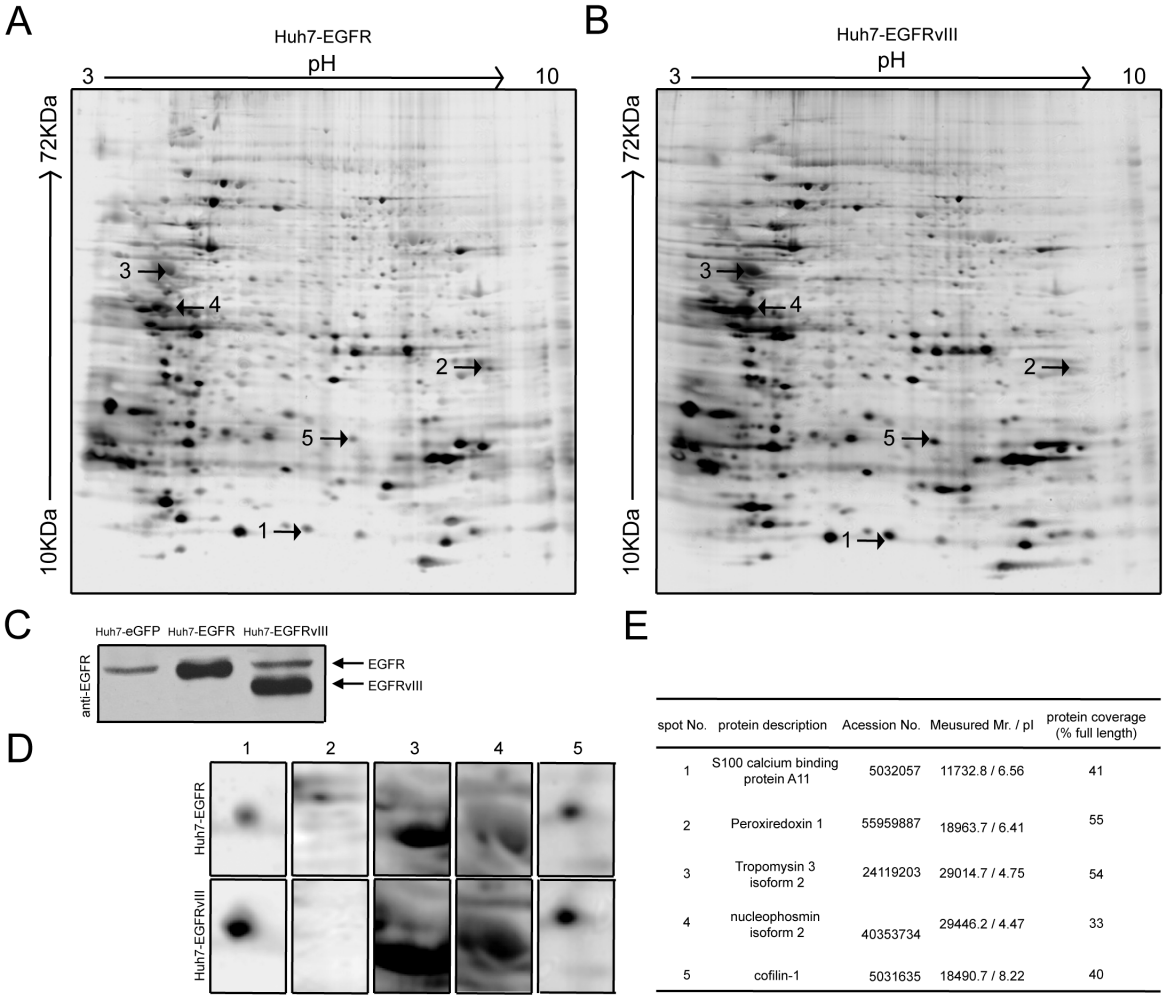
Differential protein expression between Huh7-EGFR and Huh7-EGFRvIII cells. (A) A 2-DE reference map of Huh7-EGFR cells. (B) A 2-DE reference map of Huh7-EGFRvIII cells. (C) EGFR and EGFRvIII overexpression was confirmed by Western blotting. (D) Magnified regions of the gels showing potential differential protein expression between Huh7-EGFR and Huh7-EGFRvIII cells. (E) Differentially expressed proteins identified in the MS analysis between Huh7-EGFR and Huh7-EGFRvIII cells.

Eighty micrograms of proteins from Huh7-EGFR and Huh7-EGFRvIII cells was separated by 2-DE. Scanned images of silver-stained 2-DE gels are shown in Figure 1A and B. The results were analysed using Image-Master software, revealing approximately 1005±58 protein spots in Huh7-EGFR and 936±116 spots in Huh7-EGFRvIII cells. Five of these spots were differentially expressed between these two cell lines in three independent repeated trials. The corresponding regions are enlarged and shown in Figure 1D. The five differentially expressed protein spots were further analysed by LC-MS/MS, and five candidate molecules were identified: S100 calcium binding protein A11 (S100A11), peroxiredoxin 1 (PRDX1), tropomyosin 3 isoform 2, nucleophosmin1 isoform 2, and cofilin-1 (supplemental ms data). As shown in Figure 1E, all five proteins had high sequence coverage. EGFR and EGFRvIII overexpression was confirmed by Western Blotting (Figure 1C).

### S100A11 expression is upregulated in Huh7-EGFRvIII cells

**Figure 2:**
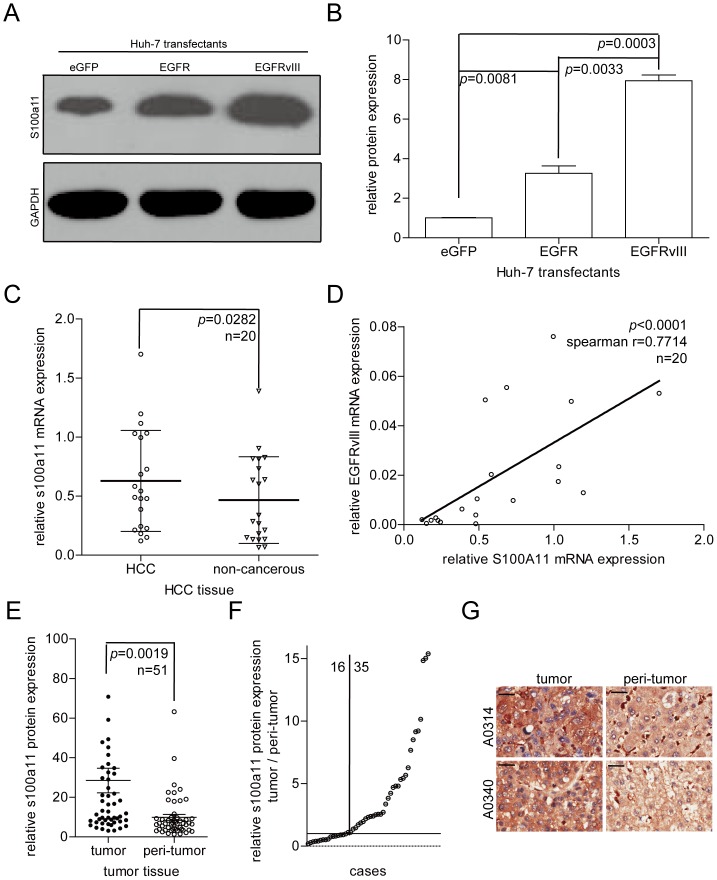
S100A11 is upregulated in EGFRvIII-positive Huh-7 cells and HCC tissues. (A) Analysis of S100A11 protein upregulation by Western Blotting in Huh7-EGFRvIII cells. (B) Quantification of S100A11 protein expression in Huh7-EGFRvIII cells. (C) S100A11 mRNA expression in 20 pairs of HCC tissues and surrounding tissues was detected by qRT-PCR. (D) The correlation between EGFRvIII mRNA expression and S100A11 mRNA expression in 20 HCC tissues (analysis by nonparametric correlation (spearman), and options with two-tailed). (E) S100A11 staining intensity is high in HCC tissues (in 51 pairs of HCC tissue arrays). (F) S100A11 staining intensity is high in 68.6% of HCC tissues (35/51). (G) Representative staining images. Black scale bars, 10 µm.

The mRNA expression levels of the five candidate molecules were further analysed by qRT-PCR. S100A11 (2.4-fold) and cofilin-1 (1.5-fold) were upregulated in Huh7-EGFRvIII cells (Figure S1 in File S1 and File S2, the other three genes showed no significant change). We confirmed that S100A11 was significantly upregulated in Huh7-EGFRvIII cells by Western Blotting (Figure 2A). The quantification results of Western Blotting further demonstrated that S100A11 was clearly upregulated in Huh7-EGFRvIII cells (Figure 2B).

We next asked whether S100A11 was also upregulated in the HCC samples. The RT-PCR results showed that S100A11 mRNA expression was upregulated by 34.8% in HCC tissue samples (relative to non-cancerous tissues) (Figure 2C, *p* = 0.0282). We also analysed the correlation between EGFRvIII mRNA expression and S100A11 mRNA expression. The data show that S100A11 mRNA expression was tightly correlated with that of EGFRvIII in HCC tissues (Figure 2D).

To further confirm the expression of S100A11, we examined S100A11 protein expression in a 51-pair tissue microarray. The data showed that the staining intensity of S100A11 was higher in HCC tissue than in the adjacent non-cancerous tissues (Figure 2E). Additionally, S100A11 staining was elevated in 68.6% (35/51) of HCC tissues (Figure 2F). A representative image of S100A11 staining is shown in Figure 2G.

These data suggest that EGFRvIII stimulates S100A11 expression, and S100A11 is highly expressed in HCC tissues.

### S100A11 promotes HCC invasion and migration

**Figure 3:**
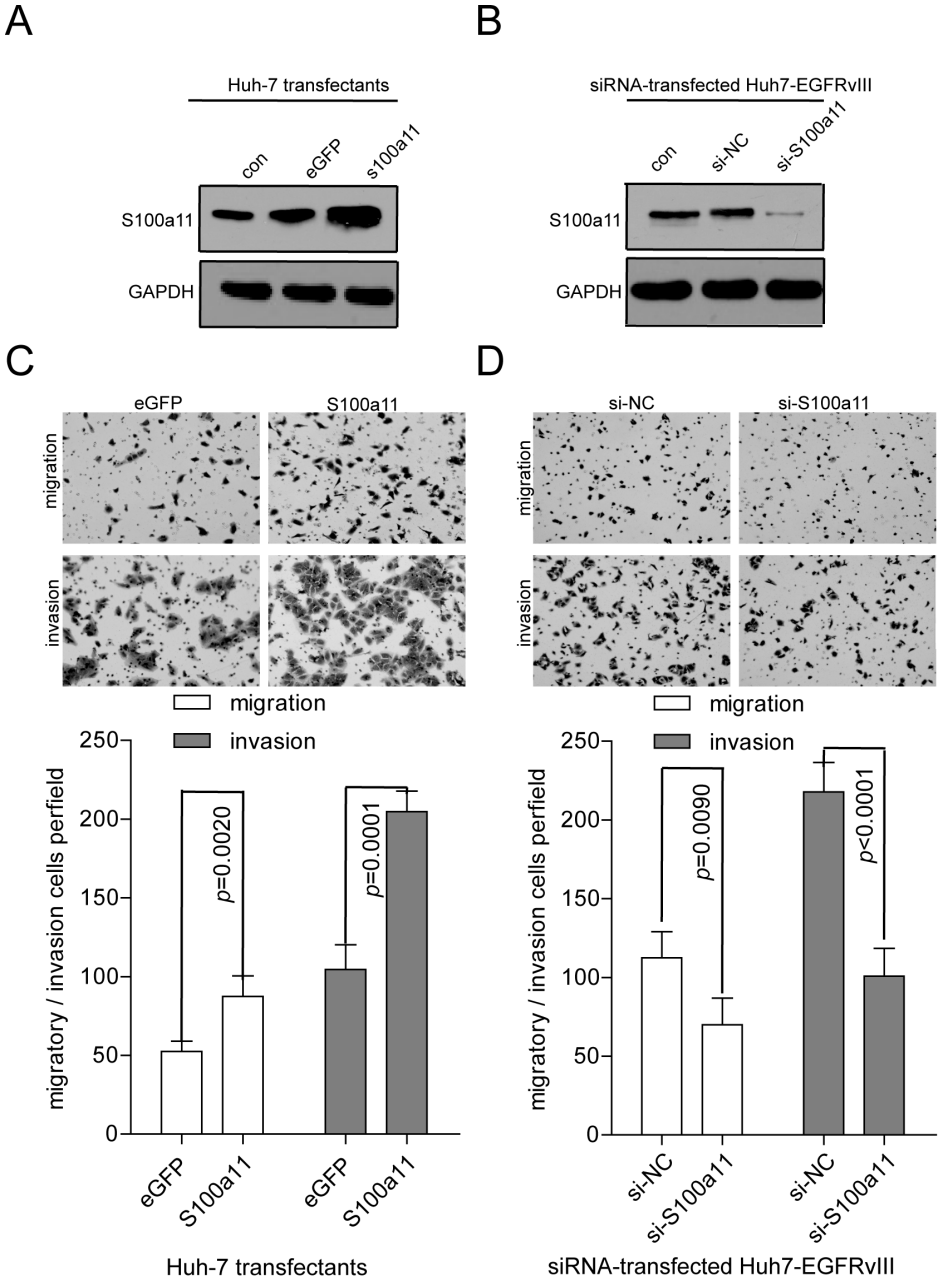
S100A11 promotes HCC cell invasion and migration. (A) Confirmation of S100A11 overexpression in Huh-7 cells by Western blotting. (B) Confirmation of S100A11 knockdown by siRNA (against S100A11) transfected into Huh7-EGFRvIII cells. (C) Overexpression of S100A11 enhanced cell migration and invasion in Huh-7 cells. (D) S100A11 knockdown inhibited cell migration and invasion in EGFRvIII-transfected Huh-7 cells.

To understand the contribution of S100A11 to EGFRvIII-mediated biological functions, the complete CDS of S100A11 was introduced into Huh-7 cells using a lentiviral expression system. The overexpression of S100A11 in Huh-7 cells was confirmed by Western Blotting (Figure 3A). An siRNA against S100A11 was used to suppress the expression of S100A11 in Huh7-EGFRvIII cells. The knockdown of S100A11 via siRNA was confirmed by Western Blotting (Figure 3B). S100A11 expression did not demonstrate an apparent effect on cell viability or cell proliferation in Huh-7 or Huh7-EGFRvIII cells relative to eGFP- or siControl-transfected cells (data not shown). Interestingly, the overexpression of S100A11 significantly promoted cell invasion (*p* = 0.0001) and migration (*p* = 0.0020) (Figure 3C). Conversely, knocking down S100A11 expression in Huh7-EGFRvIII cells significantly inhibited cell invasion (*p* = 0.0001) and migration (*p* = 0.0090) (Figure 3D). These data indicate that S100A11 can promote cell invasion and migration in EGFRvIII-transfected cells and should be an important player in EGFRvIII-mediated invasiveness.

### S100A11 promoter activity is stimulated in EGFRvIII-transfected Huh-7 cell lines

**Figure 4:**
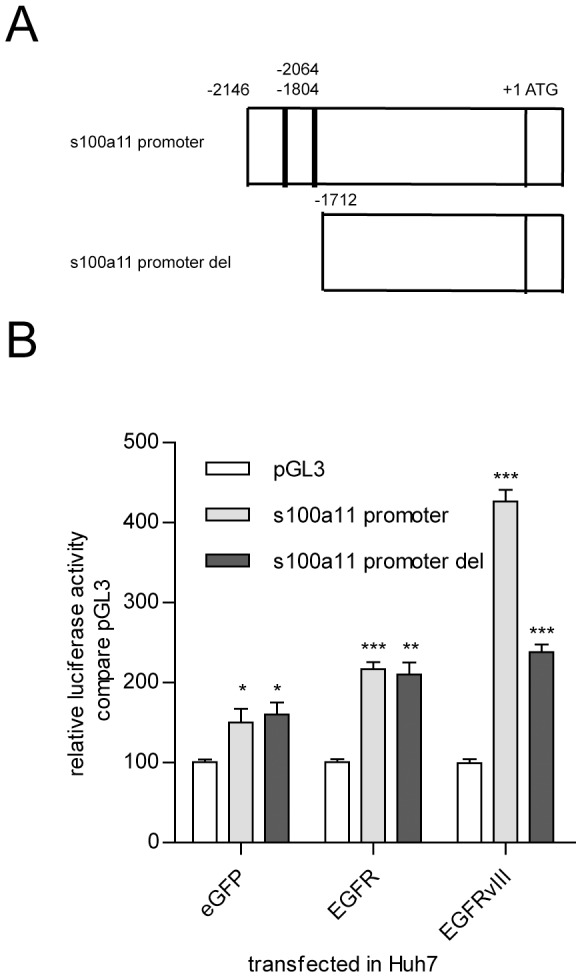
EGFRvIII stimulates S100A11 promoter activity. When the S100A11 promoter inserted into the pGL3basic vector was transfected into Huh7-EGFRvIII cells, the promoter activity was upregulated by 2.8 fold compared with transfection of the same construct into Huh7-eGFP cells (B). When the S100A11 promoter was deleted down to -1712 bp (A), this stimulation of promoter activity was abolished (B).

S100A11, a protein regulator of pseudopodial actin dynamics and stress fibre changes, is an important factor during cell migration and invasion [29]. To investigate how EGFRvIII regulates S100A11 expression, we amplified the sequence of the S100A11 promoter and detected the promoter activity in Huh-7 cells with stable overexpression of EGFRvIII/eGFP using the luciferase reporter assay. The data show that the S100A11 promoter activity was 2.8-fold upregulated in Huh7-EGFRvIII cells compared with that in Huh7-eGFP cells, and the activity was also 1.44-fold upregulated in Huh-7-EGFR cells compared with that in Huh7-eGFP cells (Figure 4B). To ascertain the enhancer that regulates the promoter activity, we deleted the promoter sequence to limit the enhancer binding region. When the promoter was deleted to −1712 (Figure 4A), the activity of the truncated promoter showed no difference between in the Huh7-EGFR and Huh7-EGFRvIII cells (Figure 4B). According to the transcription factor binding prediction (http://www.cbrc.jp/research/db/TFSEARCH.html), there are two STATx binding sites at the −2146 and −1712 regions (sequence shown in File S4). These data demonstrate that EGFRvIII could stimulate S100A11 promoter activity, and the −2146 to −1712 region is an important region in this stimulation.

### EGFRvIII promotes S100A11 expression by activating STAT3

**Figure 5:**
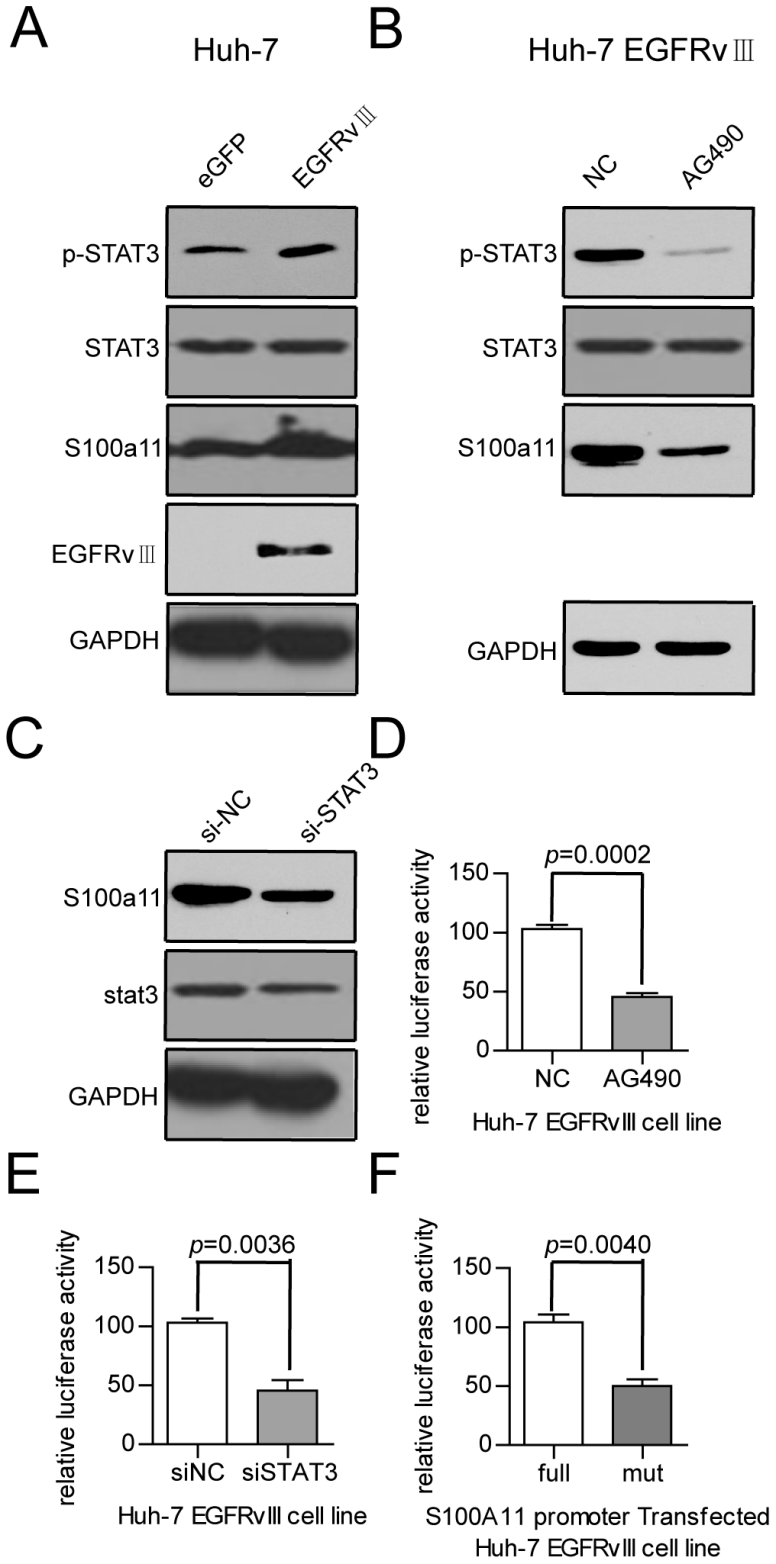
EGFRvIII enhanced S100A11 expression by stimulating STAT3 phosphorylation. (A) p-STAT3 and S100A11 expression is upregulated in Huh7-EGFRvIII cells. (B) STAT3 phosphorylation and S100A11 expression are inhibited in AG490-treated Huh7-EGFRvIII cells. (C) S100A11 expression is downregulated in STAT3-knockdown Huh7-EGFRvIII cells. (D) S100A11 promoter activity is downregulated in AG49-treated Huh7-EGFRvIII cells. (E) S100A11 promoter activity is downregulated in STAT3-knockdown Huh7-EGFRvIII cells. (F) Mutation in STATx binding sites could abolish the S100A11 promoter activity in Huh7-EGFRvIII cells.

EGFRvIII has been reported to mediate head and neck cancer cell migration and invasion by increasing STAT3 activation [26]. Additionally, we observed increased STAT3 activation in Huh7-EGFRvIII cells compared with Huh7-eGFP cells [30], and considering the prediction of two STATx binding sites at the important region (identified as −2146 to −1712), we hypothesised that STAT3 could bind to this region and regulate S100A11 expression. To test this hypothesis, we first detected the STAT3, phosphor-STAT3 and S100A11 expression levels in Huh7-EGFRvIII and Huh7-eGFP cells. The data showed that STAT3 was clearly activated in Huh7-EGFRvIII cells (Figure 5A). Next, we treated Huh7-EGFRvIII cells with AG490 (an inhibitor of the JAK/STAT3 pathway) or transfected Huh7-EGFRvIII cells with siRNA against STAT3 and found that the protein levels of S100A11 were downregulated (Figure 5B and C). Notably, Huh7-EGFRvIII cells treated with AG490 or knockdown of STAT3 could abolish the S100A11 promoter activity (stimulated by EGFRvIII, Figure 5D and E). Finally, disruption of the STAT3 binding to these two sites by mutation (sequence mutation shown in File S4) abolished luciferase activity stimulation in Huh7-EGFRvIII cells (Figure 5F). These data demonstrate that EGFRvIII promotes high S100A11 expression by activating STAT3.

## Discussion

Metastasis remains a major challenge in the treatment of patients with HCC. However, information regarding how tumour cells acquire the ability to invade through neighbouring tissue and metastasise remains somewhat limited.

EGFRvIII has been observed to be overexpressed in a wide range of cancer tissues, including HCC. However, little is known about the mechanism underlying EGFRvIII-mediated invasion. In the current study, we observed the upregulation of S100A11 in cells with transfected EGFRvIII. S100A11 promoted cell migration and invasion, and knockdown of S100A11 in EGFRvIII-transfected HCC cells could abolish EGFRvIII-promoting cell migration and invasion. Thus, S100A11 could phenocopy EGFRvIII's function in HCC.

EGFRvIII is a cancer-specific deletion mutant and has been investigated in various cancers. EGFRvIII can promote cell invasion or epithelial-mesenchymal transition in several cancer types[25], [26], [31], [32]. Previous studies have shown that EGFRvIII can promote EMT or cell invasion by activating STAT3 [33], [34] or regulating myristoylated alanine-rich protein kinase C substrate overexpression[32]. Furthermore, EGFRvIII is expressed in primary breast tumours and contributes to cancer stem cell phenotypes in breast cancer cell lines through the Wnt pathway[24]. STAT3 activation can induce EMT by targeting E-cadherin in colon cancer [33]. However, the protein effector of the EGFRvIII-STAT3 pathway in HCC is currently unknown. In the present study, we further confirmed that STAT3 could be activated by EGFRvIII, and inhibition of STAT3 activation or knock down of STAT3 could inhibit S100A11 expression and S100A11 promoter activity. Furthermore, mutation of the predicted STATx binding sites could abolish the S100A11 promoter activity stimulation by EGFRvIII. Thus, we ascertain that S100A11 is an effector in the EGFRvIII-STAT3 pathway in HCC.

S100A11 is a member of the S100 family of proteins containing two EF-hand calcium-binding motifs. This protein may function in motility, invasion, and tubulin polymerisation. S100A11 is one of the essential proteins for pseudopod protrusion and tumour cell migration and invasion. Knocking down S100A11 in metastatic cells resulted in reduced actin cytoskeleton dynamics and the induction of mesenchymal-epithelial transition (MET), which could be prevented by the stabilisation of the actin cytoskeleton[29]. S100A11 was reported to be an accurate predictor of lymph node metastases in gastric cancer [35], [36], and it was also reported to be a predictor of colon-derived liver metastases [37]. TGF-beta has been reported to induce S100A11 expression in HCC [38]. All of these studies revealed that S100A11 may be a vital protein in HCC cell invasion. However, few studies have evaluated the regulation of S100A11 expression in HCC. In the present study, we found that S100A11 is highly expressed in HCC, and the EGFRvIII-STAT3 pathway promotes the expression of S100A11.

In conclusion, the EGFRvIII-STAT3 pathway promotes S100A11 expression by enhancing S100A11 promoter activity. S100A11 is highly expressed in HCC tissues and plays a vital role in the EGFRvIII-STAT3 pathway to promote cell migration and invasion. Thus, S100A11 may serve as a valuable potential therapeutic target of HCC.

## Supporting Information

File S1 (TIF)

File S2 (DOC)

File S3 (DOC)

File S4 (DOC)
