# Supplementary figures and images for: EGFRvIII Mediates Hepatocellular Carcinoma Cell Invasion by Promoting S100 Calcium Binding Protein A11 Expression
Source: PLoS One. 2013 Dec 20;8(12):e83332. doi: 10.1371/journal.pone.0083332 (PMC3869758; doi:10.1371/journal.pone.0083332)

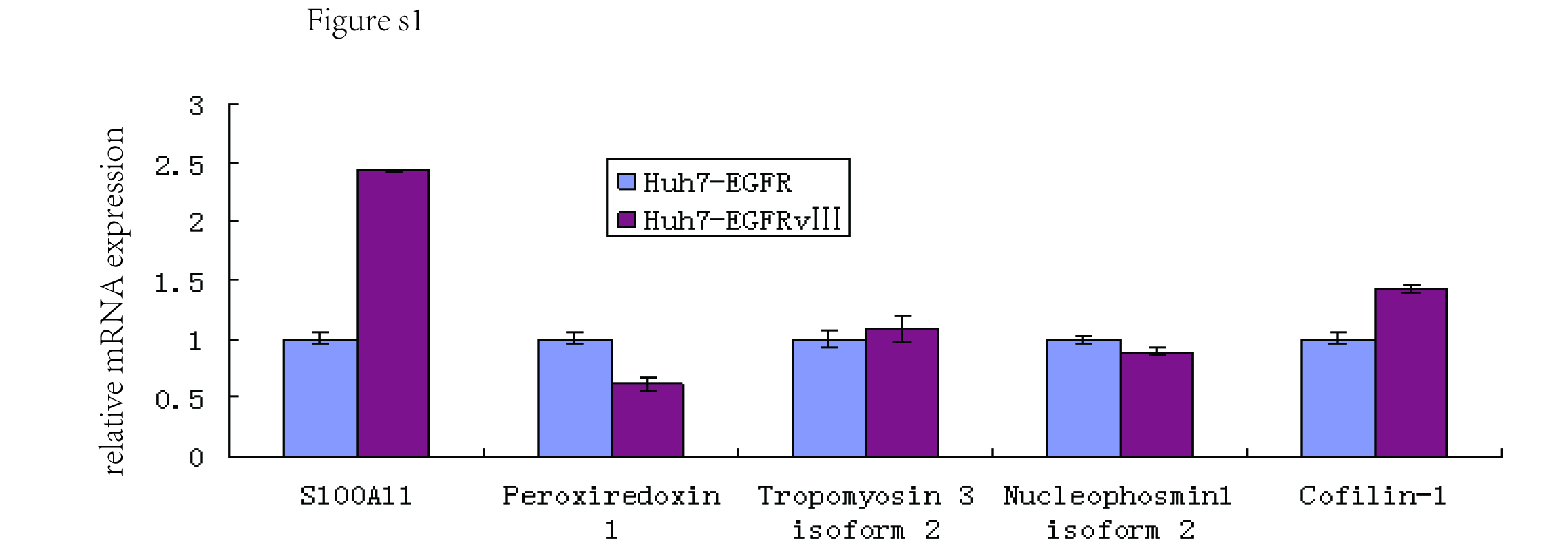

Supplement: File S1 — (TIF) [file pone.0083332.s001.tif]
