# Supplementary material for: EGFRvIII Mediates Hepatocellular Carcinoma Cell Invasion by Promoting S100 Calcium Binding Protein A11 Expression
Source: PLoS One. 2013 Dec 20;8(12):e83332. doi: 10.1371/journal.pone.0083332 (PMC3869758; doi:10.1371/journal.pone.0083332)
Supplement: File S2 — (DOC) [file pone.0083332.s002.doc]

Legend for figure s1

The mRNA expression levels of the five candidate molecules were further analysed by qRT-PCR. S100A11 (2.4-fold) and cofilin-1 (1.5-fold) were upregulated in Huh7-EGFRvIII cells compare to Huh7-EGFR cells, and the other three genes (peroxiredoxin 1 (PRDX1), tropomyosin 3 isoform 2 and nucleophosmin1 isoform 2) showed no significant change.
