# Supplementary material for: EGFRvIII Mediates Hepatocellular Carcinoma Cell Invasion by Promoting S100 Calcium Binding Protein A11 Expression
Source: PLoS One. 2013 Dec 20;8(12):e83332. doi: 10.1371/journal.pone.0083332 (PMC3869758; doi:10.1371/journal.pone.0083332)
Supplement: File S3 — (DOC) [file pone.0083332.s003.doc]

Table s1 real-time PCR for 5 candidate genes

| Gene | Direction | Primer sequences (5'-3') | Product  size (bp) |
| --- | --- | --- | --- |
| S100A11 | Sense | GAGTCCCTGATTGCTGTCTTCC | 129 |
|  | Antisense | AGGGTCCTTCTGGTTCTTTGTG |  |
| Peroxiredoxin 1 | Sense | ACCTAAGAAACAAGGAGGACTGG | 180 |
|  | Antisense | CCAACAGGGAGGTCATTTACAG |  |
| Tropomyosin3 isoform 2 | Sense | CTTGGAACGCACAGAGGAAC | 107 |
|  | Antisense | CAGCAGCACTCAGACACTTC |  |
| Nucleophosmin1 isoform 2 | Sense | GGCTTTGAAATAACACCACCAG | 125 |
|  | Antisense | TCCTCCTCCTCTTCATCTTCTG |  |
| Cofilin-1 | Sense | GACTGCCGCTATGCCCTCTATG | 147 |
|  | Antisense | CTTCTTCTTGATGGCGTCCTTG |  |
| β-actin | Sense | TCCTCCCTGGAGAAGAGCTA | 312 |
|  | Antisense | GTACTTGCGCTCAGGAGGAG |  |

Table s2 siRNA sequence

| gene | direction | sequence(5'-3') |
| --- | --- | --- |
| s100a11 | sense | CCACCUGCCAAUAGUAAU |
| stat3 | sense | AGUCAGGUUGCUGGUCAAA |
| negative control | sense | UCCUCCCUGGAGAAGAGCUA |
