# Supplementary material for: EGFRvIII Mediates Hepatocellular Carcinoma Cell Invasion by Promoting S100 Calcium Binding Protein A11 Expression
Source: PLoS One. 2013 Dec 20;8(12):e83332. doi: 10.1371/journal.pone.0083332 (PMC3869758; doi:10.1371/journal.pone.0083332)
Supplement: File S4 — (DOC) [file pone.0083332.s004.doc]

The A100A11 promoter sequence:

TGTGGTGTGTAAGGCCTCATGATTGAGTTATGGAGTAATTTAGACAAATGATTCCTTAATATGTTGGGATCATAAATTTTTCTGAGAATCAGATGAAAGATGTGGACTCTTCGCCAGAAAAATTTCTCTTATGCAAAAAATTTACATGTAACTTTAAGGTATTAAGGAACCCCAGCAGCCGTTCATGTAACACCCCCCACCACCAAAGGTTTGAAGTCTATTACCTGGCACTTGATAAGAACTGTGAGTCAAAAAGGCATTTAGACTCAGTGGCTCCAATGCTTTCAAGCAGGGAAAATGGTAGAGTGATAGTACCCTTAGCAGAGAAGACAGAAACAGGTTTGGGGAAAACTAGTTATTCCTGAATTATTGAGTCTGAAGTGCAGGTGGGACTCTCAAGTGGATTCATAAACAAGTAACTGAAAATGTGGGTCTGGAGTAGCTGAGAACTGAGATGGAGATTTGTCTCACGTGCTCAGGGCACCTGTGAGAGCAGGTGCAGGGAAGCAGTTCACACAATGCCTATAGCCCATTATCTTTCATTTGCGTCTACTCTGCAGTTTTTGGAGACCTTTCTAAGCCCTCACTTTACTTGACAGTCCTAACAATCTTATGAAACAGGAAAGGCCTATCTCAAGACCTATTACAAATAAGGAAGTGGAAGCTCAAAGGAGTGACAAGTCTGGTCAAAAGTATTGCAATGAAGTAACAGACACAAGACTGGAACCCAAGTCTTCTGATTCTTAATACAACTCCTAATTTCATTGCAAACTGCACACAGATAAATGCCTTACTAGCACGGATCAATTTTTAAGTGCAGTAGTTTGGTTTTCGGATTTTGTCTTTGTGGCTGTCCTCAGTTTACAGAAAAGTTTGAGAGAGAGGGGTGGCAATTGTCCCTTGACAATAATCCCCAGTAATCCCGTGGCAATTGATTTGGCAATACTCACCACTGTTTACTGTTAAATATCTTCCCTGTGGAGCAATAGAGAGCCCCAACTCCTAGGAACTTGTCTACAATTCTGACCCGCACTCTGTCCTAACTAACACTCTTGGTTTTGTTTTTTGTTTGTTTGTTTTTGAGATGGAGTCTTGCTCTGTCGCCCAGGCTAGAGTGCAGTGGCGCGATCTCGGCTCACTGCAAGCTCTGCCTCCGGGGTTCACGCCATTCTCCTGCCTCAGCCTCCCGAGTAGCTGGAACTACAGGCGCCCGCCACCACGTCCGGCTAATTTTTTGTATTTTTAGTAGAGACGGGGTTTTGCCGCGTTAGCCAGGATGGTCCCGATCTCCTGACCTCGTGATCCACCCGCCTCGGCCTCCCAAAGTGCTGGGATTACAGGAGTGAGCCGCTGCGCCCGGCCTGTTTTTGTTTGTTTTTTGAGACATTGTTCCGCTCTTGTTGCCCAGGCGGCTGGAGTGCAACGGCGCGATCTCAGCTCGCTGCAAACTCCACCTCCCGGGTTCAATCGATATTTCTGCCTCAGCCTCCCAAGTAGTTGGTATTACAGGCGTGTGCCACCACGCCAGGCTAATTTTGTATTTTTAGTAGAGACAGGGGTTTCACCATGTTAGTCAGGCTGATCTCAAACTCCTGACCTCATGTGATCTACCCGCCTCGGCCTCCCAAAGTGCTGGGATTACAGGCCTGAGCCACCGCGCCCGGCCCACTGCTGTTTCCAAAAGAGACCTTTTTTGCCTATCTAACCTAAATGCCCAGAATGAGCTCATACATGGGAAAGACCCACGGACATGGACAGACTTCCCGAAGACTACTGTGCAAAACTTATAATTCTAAAAAGTCAAAAATCTTCAGGAATCACAGGACGGGGTGGAGGGGGCCGGGGGGAAAGAGGAGGGAAAAGAGGGAAAGGAGGGAGAGTGAGACTGCTTAAATCGCTGGAGAGGACGGTGGGTTTATTTGAGTCTTAAAGCTCAGTTCTGTGGTTAGCGCCGGCCGCGGTCCCAGGCCCAGCCCGGGAGGCGGGGAGGGGCGGGGCAGGCGCGGAAGCTGTCTGAGTAAGGCTTGGGCAAGGCTGGGCCGGGAAGGGCGTGGGTTGAGGAGAGGCTCCAGACCCGCACGCCGCGCGCACAGAGCTCTCAGCGCCGCTCCCAGCCACAGCCTCCCGCGCCTCGCTCAGCTCCAAC***ATG***GTAAGCCTCACTTTTCTTCTTCTTCTTCTTACATGAAAAGAAAAAGAAAAGAAAAAAAACCCACATACTTCCCAGCAGACTTTATCTTTGACTTATGTGTGGACGTGGCAGGAAACAGCAGGAAAAATCAGCCTCGTGGGAGGGAACTGGGGAAGAAGGGATGGAAGGATGAAGCCCTAAAATCATCCTTGGGGTGGCCGCCTAGGGTAGCAGCGGAGGTTCTTTAGCCTTCCCAGTCACGACA

High light sequences are the STATx binding site of predicted by <http://www.cbrc.jp/research/db/TFSEARCH.html>

Mutant S100A11 promoter :

TGAGAATCA to AAAAAAAAA and TGGGGAAAA to AAAAAAAAA
